# Four new species of Andean *Pilea* (Urticaceae), with additional notes on the genus in Venezuela

**DOI:** 10.3897/phytokeys.42.8455

**Published:** 2014-10-24

**Authors:** Laurence J. Dorr, Basil Stergios

**Affiliations:** 1 Department of Botany, MRC-166, National Museum of Natural History, Smithsonian Institution, P.O. Box 37012, Washington, DC 20013-7012, USA; 2 Universidad Nacional Experimental de los Llanos Occidentales “Ezequiel Zamora” (UNELLEZ), Mesa de Cavacas, Guanare, Estado Portuguesa 3323, Venezuela

**Keywords:** Urticaceae, *Pilea*, Venezuela, Andes

## Abstract

Four new species of *Pilea* (Urticaceae) from the Andes of Venezuela are described and illustrated: *Pilea matthewii* **sp. nov.**, *Pilea miguelii* **sp. nov.**, *Pilea nicholasii* **sp. nov.**, and *Pilea nidiae* **sp. nov.** The affinities of these species and their positions within the informal classifications of *Pilea* proposed by Weddell and Killip are discussed. Notes on other species of *Pilea* found in Venezuela also are presented.

## Introduction

*Pilea* Lindl. (Urticaceae), a large genus of 700 or more species, is found worldwide in tropical, subtropical, and temperate areas although it is absent from Australia, New Zealand, and Europe (Monro et al. 2012). Southeast Asia is believed to be the center of morphological and phylogenetic diversity for the genus, while the center of species diversity is in the Caribbean and Andes (Monro 2006). Field and herbarium work focused on producing a flora of Guaramacal National Park (Portuguesa and Trujillo states), which protects part of the Venezuelan Andes, convinced us that the following four species of *Pilea* from the Andes of Venezuela should be described as new.

There has been no critical examination of the genus *Pilea* in the northern Andes (including the Coastal Cordillera of Venezuela) since Killip (1936, 1939) published his regional revision and it is not surprising that undescribed species are found. The most recent enumeration of the genus for Venezuela (Romaniuc Neto 2008) recognized 28 species of which 12 are reported from Andean states. In addition to the four species described here, we believe Romaniuc Neto (2008) overlooked two species reported from Venezuela, recognized another two species that do not occur in the country, and listed two species that might not occur in Venezuela. Thus, by our count there are at least 32 species of *Pilea* in Venezuela, the majority occurring in the Andes and the Coastal Cordillera. Our modifications to the enumeration of the species of *Pilea* known from Venezuela (Romaniuc Neto 2008) are summarized in the final section of this paper.

We are aware that the classification of *Pilea* proposed by Weddell (1869) and modified by Killip (1936, 1939) is artificial, but Killip’s informal classification especially is the only current practical way to group Andean species. A world-wide monograph of the genus is unlikely to be prepared anytime soon although Monro (2006) has proposed a phylogenetic framework for revising the genus based on *cp*DNA, *nr*DNA, and morphology. He did not find support for Weddell’s (1869) classification but did find a strong geographical signal in his molecular phylogeny. This led Monro (2006) to conclude that a combination of morphologically and geographically circumscribed groups may provide a pragmatic way to identify monophyletic units for an eventual global revision of *Pilea*.

## Methods

The new species are based principally on our collections and those of our collaborators, which were made as part of the Flora of Guaramacal project (PORT-US). We also examined collections from throughout Venezuela and adjacent Colombia that are deposited in MO, NY, PORT, US, and VEN (herbarium abbreviations follow Index Herbariorum, http://sweetgum.nybg.org/ih). The US collections were particularly useful because Killip was based at the U.S. National Herbarium (US) when he published his revisions of the Andean species of the genus (Killip 1936, 1939).

Sheet numbers are cited for the holotypes deposited in PORT. Barcodes are cited for isotypes deposited in US. Identification numbers (sheet numbers and/or barcodes) are not available for the remaining type material collected by us and our collaborators, which will only be distributed upon publication of this paper.

A morphological species concept was adopted and descriptions were modeled on those of Monro (2001, 2006) and Monro et al. (2012) in order to facilitate comparisons. Material was examined and measured using an Olympus SZH binocular microscope.

Conservation assessments were undertaken using IUCN (2001) criteria. However, the only available data for our new species concern the geographic range of these species: IUCN criteria B1 (extent of occurrence) or B2 (area of occupancy). We have no data with respect to population size or dynamics (viz., whether or not populations are declining or expanding).

## Taxonomic treatment

### Pilea matthewii

Taxon classificationPlantaeRosalesUrticaceae

Dorr & Stergios sp. nov.

urn:lsid:ipni.org:names:77142871-1

Figure 1

Pilea sp. A; Dorr et al., Contr. U.S. Natl. Herb. 40: 146. 2000 [2001].

#### Diagnosis.

*Pilea matthewii* resembles *Pilea crugeriana* Wedd. from which it differs by having simple (versus 3-rayed) foliar cystoliths and shortly pedicellate (versus sessile) staminate flowers.

#### Type.

**VENEZUELA.** Trujillo: Mpio. Boconó: Páramo de Guaramacal, SE of television towers, ca 09°14'N, 070°11'W, 2000 m, 28 Apr 1988, *L.J. Dorr et al. 4994* (holotype (♀): PORT [39536]; isotypes (♀): NY, US (excluding ♂ branchlet) [00534984], VEN).

#### Description.

Herb, 30–80 cm tall; terrestrial; dioecious. Stems erect, succulent, branched, drying dark grayish-brown or almost black, glabrous, cystoliths fusiform to elliptic or absent, internodes 7–50 × 1–3 mm (shorter and narrower distally), terete, somewhat angular in cross-section when dry. Stipules ca 0.5–1 mm long, broadly deltate, drying dark brown with lighter brown margins, persistent. Leaves petiolate, distichous; petioles at each node unequal by a ratio of 1:3–24; major petioles 3–15 (–20) mm long, canaliculate above, glabrous; minor petioles 0.5–1 mm long or subsessile, canaliculate above, glabrous; laminae at each node unequal by a ratio of 1:3.1–11.1; major laminae in a pair 2.2–11.5 × (0.8–) 1.2–2.7 cm, lanceolate or elliptic, slightly falcate, sub-chartaceous to chartaceous, 3-nerved from the base, midrib and lateral nerves prominent below, lateral nerves visible almost the entire lamina length but disappearing just below the apex, secondary nerves 8–16 pair, borne 70–80 (–90)° to the midrib and then strongly curved distally, upper surface drying dark grayish-brown or almost black, glabrous except for scattered, minute, orange-brown peltate scales, cystoliths fusiform or absent, lower surface drying dark greenish- or reddish-brown, glabrous, base slightly asymmetrical, cuneate, margin regularly toothed, apex acuminate; minor laminae in a pair 0.7–2 × 0.4–1.5 mm, ovate to broadly-ovate, base slightly asymmetrical, auriculate, apex abruptly acuminate, otherwise as major laminae. Inflorescences 8–10 per stem, unisexual; bracts ca 0.75–1 mm long; bracteoles ca 0.75 mm long. Staminate inflorescences (1) 2 per axil, 6–12 mm long, bearing 12–25 flowers in a lax cyme; peduncles 1.5–7 mm long, usually shorter than major petioles, occasionally with cystoliths and/or minute, peltate scales present, otherwise glabrous; pedicels ca 0.5 mm long, glabrous. Staminate flowers ca 1.5 × 1 mm immediately prior to anthesis, whitish-green; tepals 4, ca 1.5 mm long, occasionally cystoliths present and often minute, peltate scales present at base, otherwise glabrous, the subapical appendages unequal, ca 0.25 mm long, corniculate, glabrous; stamens 4. Pistillate inflorescences (1) 2 per axil, ca 3 mm long, bearing 10–26 flowers in a congested cyme; peduncles ca 1–15 mm long, glabrous; pedicels 0.25–1 mm long, glabrous. Pistillate flowers ca 1–1.25 mm long; cucullate tepal ca 1–1.25 mm long, ± lanceolate, appendage ca 0.25 mm long; lateral tepals ca 1–1.25 mm long, narrowly ovate. Infructescences 8–17 (–29) mm long; peduncles 5–13 (–23) mm long; achenes ca 1–1.5 × 0.5–1 mm, compressed, asymmetrically ellipsoid or lachrymiform, verrucose, margin narrowly thickened.

**Figure 1.**
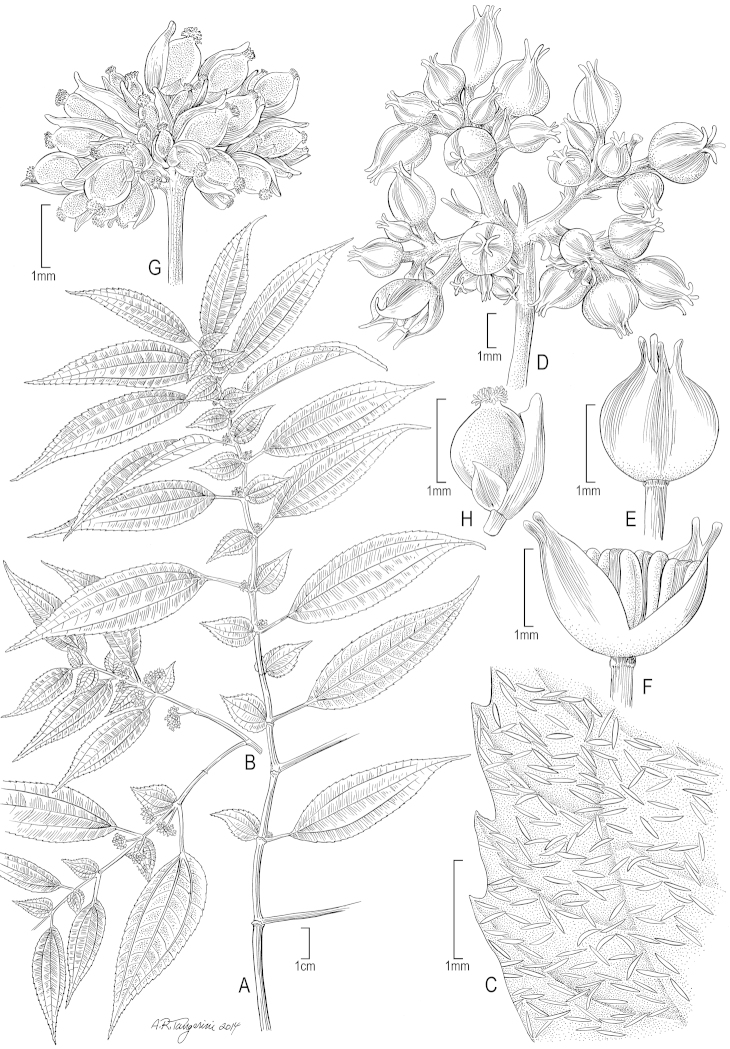
*Pilea matthewii*. **A** Habit of pistillate plant; note the sessile unequal leaf laminae at each node **B** Branchlet of staminate plant; note the unequal leaf laminae at each node **C** Leaf detail (upper surface of minor lamina) showing cystoliths **D** Staminate inflorescence **E** Staminate flower **F** Staminate flower showing anthers **G** Pistillate inflorescence **H** Pistillate flower. (**A–C, G, H** from *L.J. Dorr et al. 4994* (US); **D–F** from *B. Stergios et al. 20080* (US)).

#### Distribution and ecology.

Known only from the Andes of Venezuela (Portuguesa and Trujillo states) where it is found in the understory of cloud forest; 1000–2600 m.

#### Etymology.

The epithet recognizes Matthew Dorr who participated in a number of expeditions to Guaramacal in search of specimens for the Flora of Guaramacal project (PORT-US).

#### Specimens examined.

**VENEZUELA.** **Portuguesa:** Mpio. Sucre: Parque Nacional Guaramacal, Sector El Paramito, Camino Real Paramito – Batatal, 09°19,03'N, 070°04,25'W to 09°20,35'N, 070°04,08'W, 1550–1950 m, 17 Mar 1999, *N. Cuello et al. 1470* (PORT, US); Los Paramitos, a 20 km por aire al SO de Biscucuy, a orillas de la quebrada El Alto tambien conocida como La Lora, 09°20'N, 069°05'W, 1000–1500 m, 17 Sep 1983, *B. Stergios et al. 6340* (PORT); La Divisoria de la Concepción, 09°18'N, 070°06'W, 1700 m, 23 Oct 1985, *H. van der Werff et al. 7560* (PORT). **Trujillo:** Mpio. Boconó: linderos del Parque Nacional Guaramacal, Laguna de Agua Negra, 09°18'N, 070°10'W, 1840 m, 27 Oct 2001, *J. Angulo & J. Infante 17* (PORT); 2 km al N-O del Caserío Cerros de Guaramacal, 42 km al S-E de Boconó, ca 09°11'N, 070°10'W, 1500 m, 25 Jul 1984, *G. Aymard & F. Ortega 2903* (PORT); Limites del Páramo de Guaramacal y el bosque nublado, 25 km al S-E de Boconó, ca 09°13'N, 070°10'W, 2200–2600 m, 26 Jul 1984, *G. Aymard et al. 2954* (NY, PORT); Parque Nacional Guaramacal, Sector Las Cruces, Camino Real La Aguadita – Batatal, 09°20,11'N, 070°05,57'W, 1900–1950 m, 17 Mar 1999, *N. Cuello et al. 1486* (PORT, US), Ibid., *N. Cuello et al. 1498* (NY, PORT, US); P.N. Guaramacal, “El Campamento” below Cerro El Diablo, ca 10 km S of Boconó on road from Fundación La Salle to El Santuario, 09°09'N, 070°17'W, 1910 m, 21 Jul 1995, *L.J. Dorr et al. 8192* (G, K, NY, PORT, US, VEN); Parque Nacional Guaramacal, trail from la Laguna de las Aguas Negras to la Qda. Salvaje, N slope of mountain, 09°19'N, 070°11'W, 27 Oct 1998, *L.J. Dorr et al. 8292* (K, PORT, US); Parque Nacional Guaramacal, road from Boconó to Guaramacal, SE of Boconó, ca 15 km from the post of the park guards, 09°13'N, 070°12'W, 2 Nov 1998, *L.J. Dorr et al. 8404* (PORT-unicate), Ibid., *L.J. Dorr et al. 8407* (K, MO, PORT, US); Parque Nacional Guaramacal, trail from El Cafenol (E of Mosquey) to Fila Los Recostaderos, 1790–2200 m, 12 Jun 2001, *L.J. Dorr et al. 8924* (K, PORT, US); 12 km ESE of Boconó, 1 km N to 4 km NE of Guaramacal, 09°12’ to 09°13'N, 070°09'W, 1600–1900 m, 15 Mar 1982, *R. Liesner et al. 12947* (PORT, VEN), Ibid., *R. Liesner et al. 12998* (PORT, VEN), Ibid., *R. Liesner et al. 13019* (PORT, VEN); Parque Nacional Guaramacal, sector El Santuario, “La Punta,” 1860 m, 9–16 Jul 1998, *B. Stergios 17348* (PORT, US), Ibid., *B. Stergios 17401* (K, NY, PORT, US); Parque Nacional Guaramacal, sector El Santuario, vertiente y cresta-divisoria entre qbda. Honda y qbda. Kubiscú, 2000–2300 m, Jan 2001, *B. Stergios & R. Caracas 19064* (K, PORT, US); Parque Nacional Guaramacal, sector vertiente sur, Aug 2001, *B. Stergios & R. Caracas 19301* (PORT, US); Cerro Guaramacal, Boconó, bajando hacia el caserío de Guaramacal, 25–26 Nov 1982, *B. Stergios et al. 4700* (PORT, US); Parque Nacional Guaramacal, trail from Casa Vicuyal toward Páramo de Vicuyal, 2200–2600 m, 10 Apr 2003, *B. Stergios et al. 20080* (K, MO, NY, PORT, US); Parque Nacional Guaramacal, Casa Vicuyal, 2100 m, 12 Apr 2003, *B. Stergios et al. 20182* (K, MO, NY, PORT, US); Parque Nacional Guaramacal, SE slopes of Cerro Guaramacal on road from Boconó to Guaramacal, Qda. Pollo, 09°13'N, 070°10'W, 2200 m, 22 Sep 2003, *B. Stergios et al. 20668* (K, MO, NY, PORT, US). Parque Nacional Guaramacal, “El Campamento,” below Cerro El Diablo and vicinity, 1800–2000 m, 16–18 Aug 2005, *B. Stergios et al. 20859* (K, PORT, US).

#### Discussion.

*Pilea matthewii* belongs in the Heterophyllae species group of Weddell (1869) and the Centradenioideae species group of Killip (1936). The new species most closely resembles *Pilea crugeriana* of the Coastal Cordillera of Venezuela, but it is readily distinguished by its simple (versus 3-rayed) cystoliths and shortly pedicellate (versus sessile) staminate flowers. Other characters that separate these two species are given in Table 1.

**Table 1.** Diagnostic characters that distinguish *Pilea matthewii* and *Pilea crugeriana*.

Characters	*Pilea matthewii*	*Pilea crugeriana*
Foliar cystoliths	simple	3-rayed, rarely simple
Leaf margins	teeth sharp, apices often hyaline	teeth blunt, rarely sharp, apices never hyaline
Stipules	persistent	caducous
Staminate flower pedicels	ca 0.5 mm	sessile
Staminate tepals	appendages ca 0.25 mm	unappendaged

#### Conservation status.

Using IUCN criteria (IUCN 2001) we could not identify a threat to *Pilea matthewii*. We are aware of 15–20 distinct populations in Guaramacal National Park, which protects an area of 225 km^2^. Although this area is relatively small, the species is frequently encountered and the number of known populations exceeds the number of locations deemed critical under IUCN criterion B2(a) for either Endangered (E) or Vulnerable (VU). In addition, the east-facing slopes of the Sierra Nevada de Mérida, which have similar habitat, are very poorly collected (Dorr et al. 2005) and might harbor additional populations of this species.

### Pilea miguelii

Taxon classificationPlantaeRosalesUrticaceae

Dorr & Stergios sp. nov.

urn:lsid:ipni.org:names:77142872-1

Figure 2

Pilea sp. B, Dorr et al., Contr. U.S. Natl. Herb. 40: 146. 2000 [2001].
Pilea sp. B, vel aff., Dorr et al., Contr. U.S. Natl. Herb. 40: 146. 2000 [2001].

#### Diagnosis.

Similar to *Pilea haenkei* Killip in the extreme difference in size of leaf laminae at each node, but differing in lamina shape (narrowly ovate to ovate or obovate versus ovate-lanceolate) and base (asymmetrically cuneate versus cordate).

#### Type.

**VENEZUELA.** Trujillo: Mpio. Boconó: “Laguna Negra,” carretera entre Batatal y Mosquey, 1700 m, 3 Apr 1994, *B. Stergios & M. Niño 16028* (holotype: PORT [58122]; isotypes: NY, US [00727846]).

#### Description.

Herb or shrublet, to 1.5 m tall; terrestrial; monoecious. Stems erect, branched, ± suffruticose, drying dull green or dark blackish-brown, glabrous, cystoliths fusiform, sometimes very dense, internodes 2.3–11 cm × 2–3 mm (shorter distally), terete and becoming ± angulate in cross-section when dry. Stipules ca 2 mm long, deltoid, drying light-brown, caducous. Leaves petiolate, distichous; petioles at the same node unequal by a ratio of 1:(5–) 10–20, major petioles 0.5–4 cm long, minor petioles ca 1 mm long or subsessile, glabrous; laminae of leaves at each node unequal by a ratio of 1:5–11.5; major laminae in a pair 5–11.5 × 2–5.5 (–7) cm, narrowly ovate to ovate or obovate, slightly asymmetric, membranous, 3-nerved from the base or lateral nerves diverging from the midrib 1–2 mm above the base, secondary nerves 12–14 pair, borne 80–90° to the midrib; upper surface dull or dark green, glabrous, cystoliths fusiform, unequal in size, often dense, lower surface pale or dull green, glabrous, midrib and secondary nerves prominently raised, base asymmetrically cuneate, margin coarsely crenate to serrate its entire length, apex long acuminate; minor laminae in a pair 0.9–2 × 0.5–1 cm, otherwise as major laminae. Inflorescences > 20 per stem, unisexual, white, whitish-green or green; bracts broadly deltate, ca 1 mm long; bracteoles broadly deltate, ca 1 mm long. Staminate inflorescences 4 per axil, 10–15 × 17–20 mm, bearing > 50 flowers in a loose, spreading cyme; peduncles 2–5 mm long, glabrous; pedicels ca 0.5–1 mm long, glabrous. Staminate flowers ca 1 mm long, greenish-white; tepals 4, ca 0.75 mm long, ± verrucose; stamens 4. Pistillate inflorescences 4 per axil, ca 10 × 18 mm, bearing > 50 flowers in a loose, spreading cyme; peduncles 2–5 mm long, glabrous; pedicels 0.5–0.75 mm long, glabrous. Pistillate flowers ca 0. 5 mm long. Infructescences not seen.

**Figure 2.**
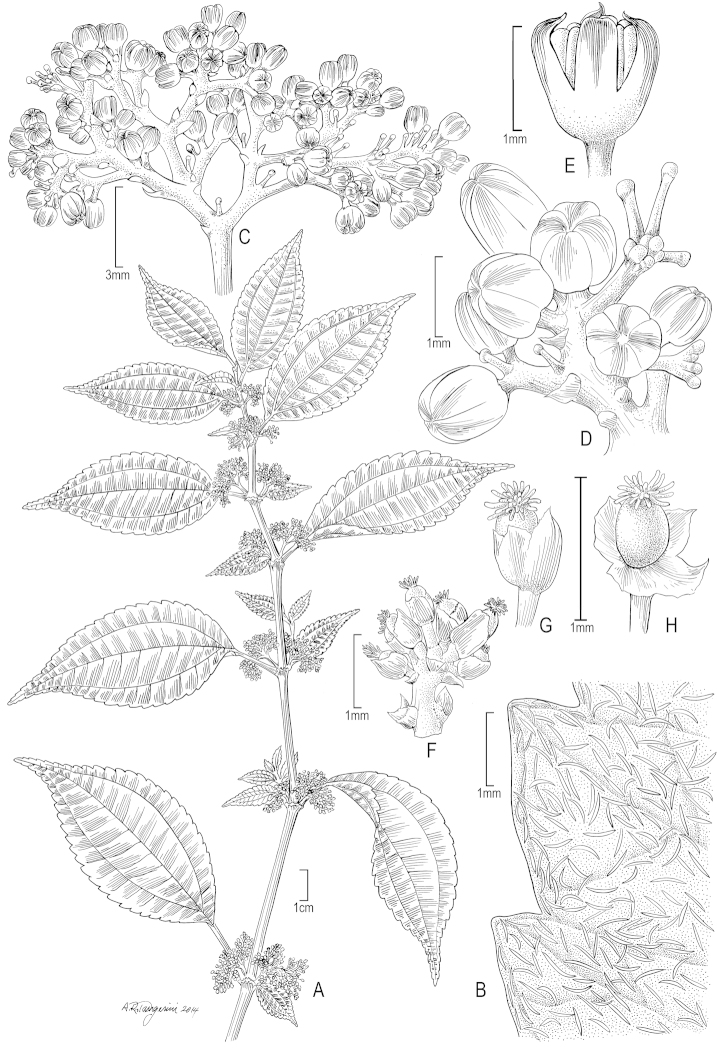
*Pilea miguelii*. **A** Habit; note the unequal leaf laminae at each node **B** Leaf detail (major lamina upper surface) showing cystoliths **C** Staminate inflorescence **D** Detail of staminate inflorescence. **E** Staminate flower showing tepals covering anthers **F** Pistillate inflorescence **G** Pistillate flower **H** Pistillate flower with tepals teased apart to show mature ovary. (**A** from *B. Stergios & M. Niño 16028* (PORT); **B–E** from *B. Stergios & M. Niño 16028* (US); **F–H** from *J.A. Steyermark 55767* (US)).

#### Distribution and ecology.

Known only from the Andes of Venezuela (Lara, Mérida, and Trujillo states) where it forms colonies in the understory of cloud forest; 1490–2600 (–3210) m.

#### Etymology.

This species is named for S. Miguel Niño, professor at UNELLEZ, Guanare, and valued collaborator in our investigations of the Andean flora.

#### Specimens examined.

**VENEZUELA.** **Lara:** Mpio. Morán: Quebrada Los Cedros (09°31'N, 070°01'W), 15 km al S de Humocaro Alto, hacia Guaitó, 1500 m, 8 Jul 1974, *J.A. Steyermark & V. Carreño Espinoza 110270* (VEN). **Mérida:** Mpio. Arzobispo Chacón: NW- and W-facing slopes of Quebrada de Montaña, in La Montaña de Los Torritos, tributary to Río Canaguá, above Finca La Montaña, 8 km SW of Canaguá, 1925–2075 m, 8 May 1944, *J.A. Steyermark 56365* (US). Mpio. Rangel: Quebrada entre Aracay y La Cuchilla, 08°56'N, 070°36'W, 2250 m, 28 Dec 1985, *A. Fernandez 1582* (PORT). Mpio. Libertador: between Los Corales and Las Cuadras, 1490–3210 m, 25 Mar 1944, *J.A. Steyermark 55767* (US). **Trujillo:** Mpio. Boconó: Límites del Páramo de Guaramacal y el bosque nublado, 25 km SE de Boconó, ca 09°13'N, 070°10'W, 2200–2600 m, 23 Jan 1986, *G. Aymard et al. 5000* (PORT); Parque Nacional Guaramacal, en los alrededores del acueducto de Boconó, detras de la Laguna de Los Cedros, 09°14'38"N, 070°13'12"W, 1850 m, 12 Mar 1998, *N. Cuello et al. 1399* (PORT, US); Parque Nacional Guaramacal, vertiente occidental, Sector El Santuario, alrededores de La Cueva, ca 09°10'N, 070°18'W, 1800 m, 1 May 1998, *N. Cuello et al. 1432* (NY, PORT, US); 13 km ESE of Boconó, 1 km W of Guaramacal, 09°11'N, 070°09'W, 1600 m, 16 Mar 1982, *R. Liesner et al. 12896* (MO, PORT, VEN). Entre Boconó y El Batatal, 1800 m, 5 Sep 1966, *J.A. Steyermark & M. Rabe 97410* (NY, US, VEN).

#### Discussion.

*Pilea miguelii* belongs in the Heterophyllae species group of Weddell (1869) and the Centradenioideae species group of Killip (1936). The new species is easily recognized by the combination of the extreme difference in leaf laminae size at each node and the branching inflorescences.

One of the specimens (*Steyermark 56365*, US) that fits our concept of *Pilea miguelii* was identified by Killip (in sched.) as *Pilea losensis* Killip, but the similarity is superficial. Killip (1936) included *Pilea losensis* in his Multiflorae species group and unlike our new species the leaf laminae at each node are ± equal in size (versus distinctly unequal), the laminae are narrowly elliptic to oblong-elliptic (versus narrowly ovate to ovate or obovate), and the apices are acuminate (versus long acuminate). In addition, the type of *Pilea losensis*, at least, is sparingly branched while most collections of *Pilea miguelii* are profusely branched.

Another specimen (*Steyermark 55767*, US) that fits our concept of *Pilea miguelii* was identified by Killip (in sched.) as *Pilea carnosula* Wedd., also in his Multiflorae species group (Killip 1936). This “new record” for Venezuela was reported by Steyermark (1957 as “*carnulosa*”) and subsequently repeated by Romaniuc Neto (2008 as “*carnulosa*”). The two species are only superficially similar. The leaf laminae at each node are ± similar in size in *Pilea carnosula* and the major laminae of *Pilea carnosula* are smaller than those of *Pilea miguelii* (0.8–4 versus 5–11.5 cm long).

The very dense, whitish covering of cystoliths on the leaves and stems of one collection (*Steyermark & Rabe 97410*) from Trujillo makes the material look different than the type of *Pilea miguelii*, but a careful examination of other morphological characters (leaf size, shape, venation, etc.) convinces us that this sterile collection belongs with *Pilea miguelii* as does another sterile collection (*A. Fernandez 1582*) from Mérida that also has a dense covering of cystoliths.

Table 2 summarizes the differences between *Pilea miguelii* and other species to which it is compared (see diagnosis) or with which it has been confused.

**Table 2.** Diagnostic characters that distinguish *Pilea miguelii* and several similar species.

Characters	*Pilea miguelii*	*Pilea carnosula*	*Pilea haenkei*	*Pilea losensis*
Leaf laminae at a node	unequal	± equal	unequal	± equal
Leaf shape	narrowly ovate to ovate or obovate	narrowly lanceolate	ovate-lanceolate	narrowly elliptic to oblong-elliptic
Major lamina size	5–11.5 × 2–5.5 (–7) cm	0.8–4 × 0.5–1.5 cm	9–13 × 3.5–4.5 cm	6–12 × 2–2.5 cm
Leaf base	asymmetrically cuneate	indistinctly cordate	indistinctly cordate	indistinctly cordate
Leaf apex	long acuminate	long acuminate	acuminate	acuminate

#### Conservation status.

We cannot discern a threat to *Pilea miguelii* using IUCN criteria (IUCN 2001). We are aware of 15–20 distinct populations, all but one of which is in either Guaramacal National Park or the Sierra Nevada National Park. The extent of occurrence (EOO) is less than 5000 km^2^ and the area of occupancy (AOO) is less than 500 km^2^, which might suggest that the species is Endangered (E) under criteria B1 or B2, but there are > 5 populations and no evidence of their decline.

### Pilea nicholasii

Taxon classificationPlantaeRosalesUrticaceae

Dorr & Stergios sp. nov.

urn:lsid:ipni.org:names:77142873-1

Figure 3

Pilea sp. C, Dorr et al., Contr. U.S. Natl. Herb. 40: 147. 2000 [2001].

#### Diagnosis.

Most similar to *Pilea hydrocotyliflora* Killip from which it can be distinguished by the distinctly asymmetrical laminae that are pruinose (i.e., with a waxy, powdery, whitish bloom) below.

#### Type.

**VENEZUELA.** Trujillo: Mpio. Boconó: Parque Nacional Guaramacal, Laguna de Agua Negra – parte alto [sic] de la Qda. Salvaje, 2000–2100 m, 14 Apr 2003, *B. Stergios & L.J. Dorr 20208* (holotype: PORT [86924]; isotypes: BM, G, K, MO, NY, P, US [00728426]).

#### Description.

Herb, to 50 cm tall; terrestrial or hemiepiphytic; monoecious. Stems erect, ascending or spreading, rarely trailing, branched or not, succulent, drying brown or dark reddish-brown, glabrous, younger stems often with minute peltate glands, cystoliths fusiform or absent, internodes 6–50 × ca 1–3 mm (shorter distally), terete, ± flattened when dry, fragrant when crushed (fide *Licata & Culleo 233*). Stipules ca 1–1.25 mm long, broadly deltate, drying dark brown, persistent. Leaves petiolate, distichous; petioles at each node unequal by a ratio of 1:4.3–17 (–33); major petioles 12–33 mm long, canaliculate above, glabrous; minor petioles 1–4 mm long or subsessile, canaliculate above, glabrous; laminae of leaves at each node unequal by a ratio of 1:1.2–3.2; major laminae in a pair 3.7–9 × 1.4–3.2 cm (laminae usually larger distally), ovate or obovate, asymmetrical, subcoriaceous, 3-nerved from the base or lateral nerves diverging from midrib 1–2 mm above the base, sometimes forming flap-like domatia where the 3 nerves join, midrib and lateral nerves prominent or not, lateral nerves visible almost the entire length but disappearing just below the apex, secondary nerves 6–9 (–20) pair, often becoming obscure or fading distally, borne 60–80 (–90)° to the midrib, often strongly curved distally, upper surface dark green, drying dark brown or reddish-brown, glabrous or with minute, peltate scales, cystoliths fusiform, varying in length, lower surface pruinose, pale green, drying whitish with scattered dark spots and minute, peltate scales, cystoliths sometimes present, base cuneate or less commonly truncate, asymmetrical, margin regularly toothed, sometimes teeth overlapping the lamina, apex acute to shortly acuminate, sometimes asymmetrical; minor laminae in a pair 1.4–3.5 × 0.8–1.6 mm, otherwise as major laminae. Inflorescences 1–5 per stem, unisexual; bracts ca 1 mm long; bracteoles ca 1 mm long. Staminate inflorescences 1 per axil, 33–50 mm long, bearing (18–) 40–60 flowers in a ± compact to loose cyme; peduncles 25–45 mm long, equal to or exceeding major petioles in length, glabrous except for minute, peltate scales, occasionally cystoliths present; pedicels 0.5–1.25 mm long, glabrous. Staminate flowers ca 1 × 1.5 mm immediately prior to anthesis, white, creamy-white, greenish-white or greenish-red; tepals 4, ca 1 mm long, glabrous, occasionally cystoliths present and also often minute, peltate scales, apices ca 0.25 mm long, glabrous; stamens 4. Pistillate inflorescences 1 per axil, 1–12 mm long, bearing 15–30 flowers in a ± compact head-like cyme; peduncles 0.5–8 mm long, glabrous; pedicels ca 0.25–1 mm long, glabrous. Pistillate flowers ca 1.25 mm long, cucullate tepal ca 1 mm long, elliptic or ovate, lateral tepals minute. Infructescences 23–28 mm long; peduncles 19–25 mm long; achenes 1–1.5 × ca 1 mm, slightly compressed, ± ellipsoid, verrucose, margin narrowly thickened.

**Figure 3.**
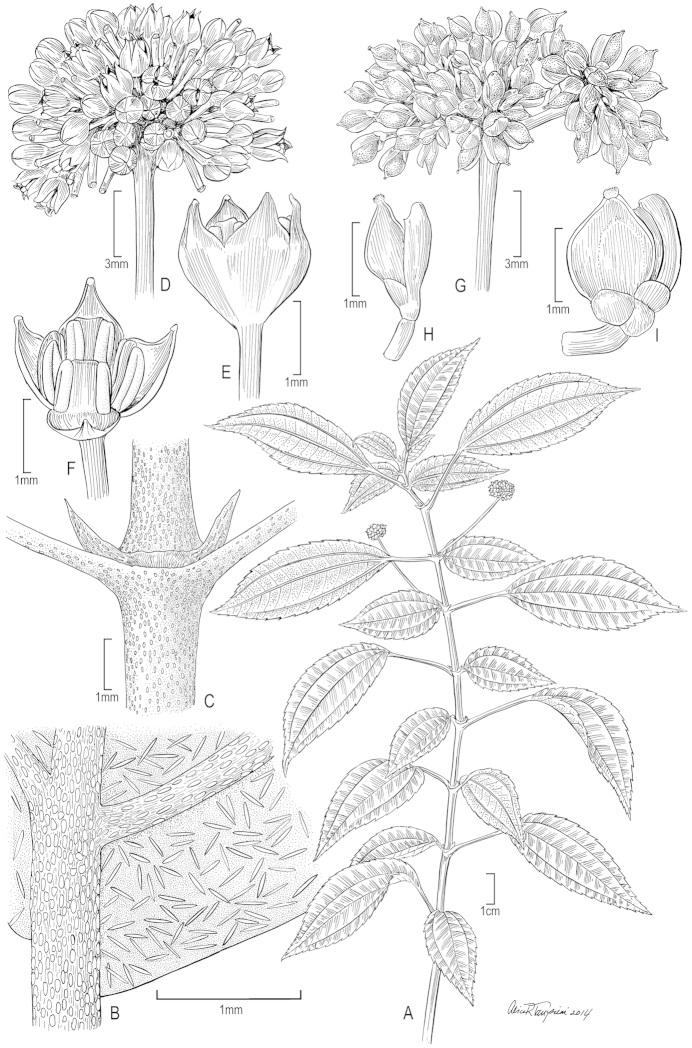
*Pilea nicholasii*. **A** Habit; note the unequal leaf laminae at each node **B** Leaf detail (major lamina upper surface) showing cystoliths **C** Stipules, stem, and petiole bases with cystoliths **D** Staminate inflorescence **E** Staminate flower ± in bud **F** Staminate flower showing stamens **G** Infructescence **H, I** Pistillate flowers with developing achenes. (**A, D–F** from *B. Stergios et al. 20074* (US); **B, C** from *B. Stergios & R. Caracas 19671* (US); **G–I** from *B. Stergios 19986* (US)).

#### Distribution and ecology.

Known only from the Andes of Venezuela (Lara, Portuguesa, and Trujillo states) where it is found in the understory of montane and cloud forest; 1900–2800 m.

#### Etymology.

This species is named for Nicholas Dorr who assisted with field work in the Venezuelan Andes, but clearly prefers the rigors of Chichiriviche to those of the mountains.

#### Specimens examined.

**VENEZUELA.** **Lara:** Mpio. Morán: SW-facing slopes at Los Aposentos, above Las Sabanetas, above Humocaro Bajo, 2500–2530 m, 3 Feb 1944, *J.A. Steyermark 55213* (NY, US, VEN); Pica que va desde Buenos Aires a Páramo Las Rosas, 09°34'N, 070°06'W, 2300–2600 m, 15 Nov 1984, *H. van der Werff & R. Rivero 7963* (PORT). **Portuguesa:** Mpio. Sucre: Fila del Helechal, en el límite con el Edo. Lara, 80 km al NO de Guanare, al N de Chabasquén, ca 2000 m, 09°32'N, 069°58'30”W, 9 Feb 1984, *B. Stergios et al. 6722* (PORT, US). **Trujillo:** Mpio. Boconó: Guaramacal, 20 km al E de Boconó, ca 09°14'N, 070°11'W, 1900–2300 m, 7 Feb 1987, *G. Aymard et al. 5226* (PORT); Parque Nacional Guaramacal, vertiente sur, ca 09°12'45'N, 070°09'51”W, 2350 m, 21 Apr 1998, *N. Cuello et al. 1416* (NY, PORT, US); Parque Nacional Guaramacal, trail from la Laguna de las Aguas Negras to la Qda. Salvaje, N slope of mountain, 09°19'N, 070°11'W, 27 Oct 1998, *L.J. Dorr et al. 8279* (PORT, US); Parque Nacional Guaramacal, road from Boconó to Guaramacal, SE of Boconó, ca 15 km from the post of the park guards, S slope of mountain, 09°13'N, 070°12'W, 3 Nov 1998, *L.J. Dorr et al. 8455* (K, MO, PORT, US), Ibid., *L.J. Dorr et al. 8471* (G, K, MO, P, PORT, US); Parque Nacional Guaramacal, trail from El Cafenol (E of Mosquey) to Fila Los Recostaderos, 1790–2200 m, 12 Jun 2001, *L.J. Dorr et al. 8872* (G, K, MO, P, PORT, US); Parque Nacional Guaramacal, en la vertiente norte, 2300 m, 27 May 1995, *A. Licata & N. Cuello 158* (PORT, US), Ibid., 09°14'59.78”N, 070°12'43.36”W, 2100 m, 19 Jun 1995, *A. Licata & N. Cuello 233* (PORT, US); Camino al Cerro Guaramacal via la laguna de “Los Cedros,” 21 Mar 1981, *B. Stergios 2544* (PORT); P.N. Guaramacal, vertiente norte, 2100 m, Mar 2003, *B. Stergios 19986* (PORT, US); Parque Nacional Guaramacal, sector trocha Laguna Negra – quebrada del Salvaje, 1850–2100 m, 15 Jun 2002, *B. Stergios & R. Caracas 19671* (MO, PORT, US); Fila de Agua Fria, 09°16.70'N, 070°8.65'W, 2700–2800 m, Jan–Feb 1996, *B. Stergios & L. Zambrano 17701* (PORT, US); Cerro Guaramacal, Boconó, 09°15'N, 070°13'W, ca 2000 m, 29 Nov 1983, *B. Stergios et al. 6561* (NY, PORT); Parque Nacional Guaramacal, trail from Casa Vicuyal toward Páramo de Vicuyal, 2200–2600 m, 10 Apr 2003, *B. Stergios et al. 20074* (G, K, MO, NY, PORT, US); Parque Nacional Guaramacal, NE slopes of Cerro Guaramacal between Laguna de Los Cedros and the summit of the road to Guaramacal, 09°15'N, 070°125'W, 21 Sep 2003, *B. Stergios et al. 20639* (PORT, US). Mpio. Carache: Entre La Peña y Agua de Obispo, 22–28 km de Carache, 2400–2500 m, 1 Mar 1971, *J.A. Steyermark 104972* (US-2 sheets).

#### Discussion.

The majority of collections of *Pilea nicholasii* have either staminate or pistillate inflorescences on a stem. Several collections, including the type (*Stergios & Dorr 20208*), however, have both staminate and pistillate inflorescences on the same stem, and at least one collection (*Cuello et al. 1416*) has both staminate and pistillate inflorescences arising from the same leaf axil. This suggests to us that the species is monoecious rather than dioecious.

Sometimes the pedicels on staminate inflorescences are sterile. The cause of this is not clear: it may be that some male flowers are caducous or, as suggested by one of the reviewers of this manuscript, the consequence of fungal infection. A number of the pistillate inflorescences, especially on specimens with conspicuous staminate inflorescences, are very cryptic with very short peduncles. Other pistillate inflorescences have pronounced peduncles. In any case, there appears to be a bias toward collecting specimens with either staminate inflorescences or infructescences probably because these plants are more visible and manifestly fertile.

*Pilea nicholasii* belongs in the Heterophyllae species group of Weddell (1869). Its placement in one of the species groups proposed by Killip (1936) is somewhat problematic as depending upon which pair of leaves at a single node are measured *Pilea nicholasii* falls into either Killip’s Centradenioideae species group with major leaf laminae more than twice as long as minor leaf laminae or his Capitellatae species group with the major leaf laminae less than twice as long as the minor ones. Among species placed in the former group, *Pilea nicholasii* is similar to *Pilea hydrocotyliflora* Killip, which was described from Colombia (Norte de Santander). However, the undersurface of the laminae is pruinose in the former and glabrous in the latter species. This makes the leaves of *Pilea nicholasii* look lighter below than above while those of *Pilea hydrocotyliflora* are uniformly green. In addition, the major laminae of the former are markedly asymmetrical whereas in the latter they appear to be ± symmetrical.

*Pilea nicholasii* also bears a superficial resemblance to *Pilea pichisana* Killip, another species in the Centradenioideae group that is known only from Peru (Junín). The major leaf laminae of *Pilea pichisana*, however, are smaller than those of *Pilea nicholasii* (2–2.8 × 1–1.3 versus 7–9 × 1.4–3.2 cm) and the cystoliths are different (punctiform versus fusiform).

*Pilea nicholasii* does not appear to have any close allies in the Capitellatae species group of Killip (1936). It keys to a group of three species that are monoecious, but none of these three species has the pruinose undersurface of the leaf laminae found in our new species.

Characters for distinguishing *Pilea nicholasii* from *Pilea hydrocotyliflora* and *Pilea pichisana* are given in Table 3.

**Table 3.** Diagnostic characters that distinguish *Pilea nicholasii* and two similar species.

Characters	*Pilea nicholasii*	*Pilea hydrocotyliflora*	*Pilea pichisana*
Leaf symmetry	asymmetrical	± symmetrical	symmetrical to asymmetrical
Major lamina size	7–9 × 1.4–3.2 cm	4–8 × 1.5–2.5 cm	2–2.8 × 1–1.3 cm
Leaf base	cuneate or less commonly truncate	subrounded	rounded or subacute
Leaf apex	acute to shortly acuminate	long acuminate	acute or acuminate
Foliar indument	pruinose	glabrous	glabrous
Foliar cystoliths	fusiform	fusiform	punctiform

#### Conservation status.

Using IUCN criteria (IUCN 2001) we could not identify a threat to *Pilea nicholasii*. We are aware of 15–20 distinct populations, the majority of which are in Guaramacal National Park. The extent of occurrence (EOO) is less than 5000 km^2^ and the area of occupancy (AOO) is less than 500 km^2^, which might suggest that the species is Endangered (E) under IUCN criteria B1 or B2, but there are > 5 populations and as with *Pilea matthewii* we would expect the species to be found in similar habitat along the east-facing slopes of the Sierra Nevada de Mérida.

### Pilea nidiae

Taxon classificationPlantaeRosalesUrticaceae

Dorr & Stergios sp. nov.

urn:lsid:ipni.org:names:77142874-1

Figure 4

Pilea sp. D, Dorr et al., Contr. U.S. Natl. Herb. 40: 147. 2000 [2001].

#### Diagnosis.

Similar to *Pilea flexuosa* Wedd. from which it differs by its asymmetrically elliptic to narrowly-elliptic or obovate (versus broadly ovate) laminae that are asymmetrically cuneate (versus rounded or cordate) at the base.

#### Type.

**VENEZUELA.** Trujillo: Mpio. Boconó: Parque Nacional Guaramacal, sector vertiente sur, 2800–2900 m, 25–30 Jul 2002, *B. Stergios & R. Caracas 19810* (holotype: PORT [85861]: isotypes: K, MO, US [00772535]).

#### Description.

Herb, to 1.25 m tall; terrestrial; dioecious. Stems erect or prostrate (fide *Stergios & Caracas 19810*), succulent, branched, drying reddish-brown, dull purple (fide *Steyermark 55533*) or almost black, glabrous, cystoliths punctiform or short fusiform, often clustered at nodes, internodes 0.8–3.5 × 1–4 mm (shorter distally), terete in cross-section, angulate when dry, nodes constricted (at least when dry). Stipules 6–11 mm long, narrowly triangular, drying pale brown or tan, persistent. Leaves petiolate, distichous; petioles at the same node unequal by a ratio of 1:11.5–13.5 (–23), canaliculate above, glabrous; major petioles 2.3–2.7 cm long; minor petioles ca 1–2 mm long; laminae of leaves at each node unequal by a ratio of 1:1.7–2.2, major laminae in a pair 6.5–9.5 × 1.5–3.2 cm, asymmetrically elliptic to narrowly-elliptic or obovate, membranous, 3-nerved with lateral nerves diverging from midrib 1–6 mm above the base, forming pocket domatia where the 3 nerves join, midrib and lateral nerves prominent below, slightly impressed (or not) above, lateral nerves visible almost the entire lamina length but disappearing below the apex, secondary nerves 8–14 pair, borne 70–90° to the midrib and then curved distally, upper surface dark green, drying dark brown, glabrous except for scattered, minute peltate scales, cystoliths fusiform or absent, lower surface pale green drying dark brown, glabrous except for scattered, minute peltate scales, base cuneate, asymmetrical, margin coarsely toothed entire length, apex long acuminate; minor laminae in a pair 3–5 × 0.8–1.5 cm, otherwise as major laminae. Inflorescences 1–10 per stem, unisexual, green suffused with maroon; bracts ca 2 mm long; bracteoles ca 1 mm long. Staminate inflorescences 1 per axil, 2.8–3.5 cm long, bearing ca 50 flowers in a compact head-like cyme; peduncles 2–3 cm long, glabrous with minute, scattered peltate scales, occasionally cystoliths present; pedicels ca 0.25 mm long. Staminate flowers ca 1.5 × 1–1.25 mm (mature flowers not seen); tepals 4, ca 2 mm long, notched inside; stamens 4. Pistillate inflorescences 1 or 2 per axil, ca 5 mm long, bearing ca 50 flowers in a ± loose cyme; peduncles ca 2 mm long, glabrous; pedicels minute. Pistillate flowers ca 0.5–0.75 mm long, cucullate tepal ca 0.5 mm long, ± lanceolate; lateral tepals minute. Infructescences 1–2.5 cm long, frequently including receptive pistillate flowers; peduncles 0.7–1.8 cm long; achenes ca 1.25 × 1 mm, compressed, asymmetrically ellipsoid or lachrymiform, verrucose, margin narrowly thickened with a very narrow hyaline wing.

**Figure 4.**
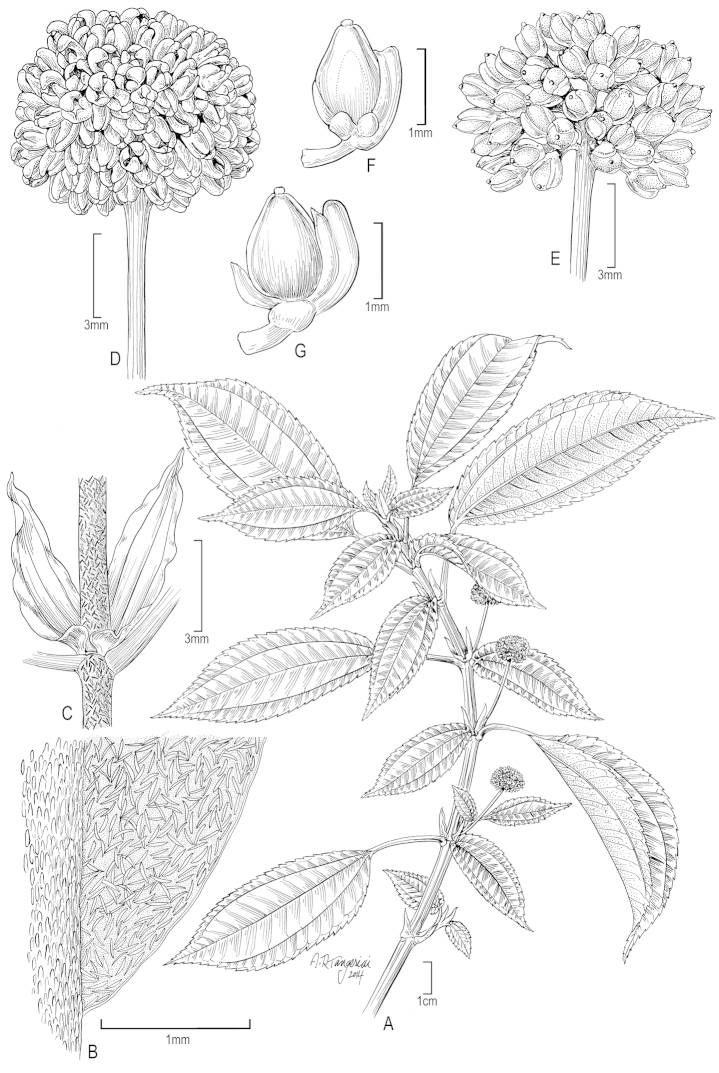
*Pilea nidiae*. **A** Habit; note the unequal leaf laminae at each node **B** Leaf detail (major lamina upper surface) showing cystoliths **C** Stipules and stem covered with cystoliths **D** Staminate inflorescence **E** Infructescence **F, G** Pistillate flowers with developing achenes. (**A, D** from *J.L. Luteyn & E. Cotton 9705* (NY); **B, C** from *J.A. Steyermark 55533* (US); **E–G** from *B. Stergios & R. Caracas 19810* (US)).

#### Distribution and ecology.

Known only from the Andes of Venezuela (Lara and Trujillo states) where it is found in the moist, shaded understory of montane forest; 2285–2900 m.

#### Etymology.

This species is named in honor of Nidia Cuello, Director of Herbario PORT, UNELLEZ, Guanare, and expert on the vegetative ecology of Guaramacal National Park.

#### Specimens examined.

**VENEZUELA.** **Lara:** Mpio. Morán: Trail from Humocaro to Buenos Aires (caserío) below Páramo Los Rosas (ca 09°40'N, 070°05'W), 2750 m, 25 Jul 1979, *R. Liesner et al. 8158* (MO, VEN); Between Buenos Aires to Canyon of El Callado, above Humocaro Alto, 2285–2740 m, 12 Feb 1944, *J.A. Steyermark 55533* (NY, US). **Trujillo:** Mpio. Boconó: Páramo Guaramacal, 20–21 km beyond jct. with hwy. NE of Boconó, ca 09°13'N, 070°13'W, 2640–2700 m, 14 Mar 1984, *J.L Luteyn & E. Cotton 9705* (MO, NY, PORT, VEN).

#### Discussion.

*Pilea nidiae* belongs in the Heterophyllae group of Weddell (1869). Its 3-nerved, toothed leaves that are unequal in size at each node and conspicuous, persistent stipules place it in the Flexuosae group of Killip (1936, 1939). We have not encountered any other Andean species from Venezuela with stipules that are as large as those of *Pilea nidiae*.

The leaf laminae of one of the paratype collections (*Steyermark 55533*) are narrower than in the type of *Pilea nidiae* and densely covered in cystoliths above and below (the type mostly lacks cystoliths). All other characters (leaf shape, venation, toothing, etc.) agree with our concept of this new species.

**Table 4.** Diagnostic characters that distinguish *Pilea nidiae* and *Pilea flexuosa*.

Characters	*Pilea nidiae*	*Pilea flexuosa*
Stipule shape (length)	narrowly triangular (6–11 mm)	ovate orbicular (4–6 mm)
Major leaf lamina size	6.5–9.5 × 1.5–3.2 cm	2–6 ×1.5–4 cm
Leaf shape	elliptic to narrowly elliptic or obovate	broadly ovate
Leaf base	cuneate	rounded or cordate
Leaf apex	long acuminate	abruptly acute to acuminate

#### Conservation status.

Using IUCN criteria (IUCN 2001) we tentatively consider *Pilea nidiae* to be Endangered (E). The known range of the species is less than 5000 km^2^ (IUCN criterion B1) and there are only four known populations and of these only two are in a protected area (IUCN criterion B1(a)). We know nothing, however, about the dynamics of these populations and whether or not they are declining.

### Additional Notes on *Pilea* in Venezuela

The following list includes corrections, additions, and deletions to the checklist of *Pilea* published in the catalog of the vascular flora of Venezuela (Romaniuc Neto 2008).

***Pilea acuminata*** Liebm. – This species should be added to the flora of Venezuela. Our voucher is: Trujillo: Mpio. Boconó: 13 km ESE of Boconó, 1 km W of Guaramacal, 09°11'N, 070°09'2, 1600 m, 16 Mar 1982, *R. Liesner et al. 12884* (PORT). Earlier Dorr et al. (2000) reported this collection as *Pilea pubescens* Liebm., with which it is allied, but from which it can be separated by the coarse serrations on the leaf margin. Also, according to Killip (1939) *Pilea pubescens* is a species found at low elevations in South America.

***Pilea arguta*** (Kunth) Wedd. – The authorship as given by Romaniuc Neto (2008) is corrected. Also, Killip (1939) expressed doubt as to whether or not this species occurs in Venezuela. The type locality is “prope Nova Valencia Caracasarum,” but the species is otherwise known only from high elevations in Colombia and Ecuador and it has not been recollected near Valencia, Carabobo nor anywhere else along the Coastal Cordillera of Venezuela.

***Pilea carnosula*** Wedd. – This species should be deleted from the flora of Venezuela because the published record (Steyermark 1957; Romaniuc Neto 2008) of its occurrence is based on a misidentification of a specimen (*Steyermark 55767*, US) here considered to be *Pilea miguelii* (see above).

***Pilea centradenioides*** Seem. – Although Romaniuc Neto (2008) stated that this species occurred in the Distrito Federal, it was not reported from Venezuela by Killip (1936, 1939). The Coastal Cordillera record conflicts with what otherwise appears to be a range corresponding to the Chocó in Central and South America. Consequently, we suspect the Venezuelan record is based on misidentification of material that might represent *Pilea crugeriana*.

***Pilea dauciodora*** Pav. ex Wedd. – Dorr et al. (2000) reported this species from Trujillo, a state record overlooked by Romaniuc Neto (2008). Lara also should be added to the distribution given by Romaniuc Neto (2008). The following collections serve as vouchers: Lara: Mpio. Morán: Parque Nacional Dinira, Páramo de Jabón, 09°34'N, 070°06'W, 2900 m, 29 Dec 1999, *R. Riina et al. 911* (US); S- and SW-facing slopes at Palojosco above Los Aposentos, above Humocaro Bajo, 2530–2375 m, 4 Feb 1944, *J.A. Steyermark 55233* (US).

***Pilea fallax*** Wedd. – Dorr et al. (2000) reported this species from Trujillo, a state record overlooked by Romaniuc Neto (2008). In addition, the authorship cited by Romaniuc Neto (2008) is here corrected.

***Pilea hyalina*** Fenzl – Killip (1939) reported this species from Aragua, a state record overlooked by Romaniuc Neto (2008). Miranda and Zulia also should be added to its Venezuelan distribution. Our vouchers are: Miranda: Mpio. Sucre: Parque Nacional El Avila ruta entre Puesto de Guarda Parques Sabas Nieves y Quebrada Chacaíto, 10°32'N, 066°51'W, 1000–1500 m, Oct 1992, *A. Fernández 8321* (US). Zulia: Machiques de Perijá: Sierra de Perijá, faldas inferiores, a lo largo del Río Yasa, vecinidad de «Guasáma,» arriba de «Kasmera» (Estación Biológica de la Universidad del Zulia), al Suroeste de Machiques, 500–600 m, 26–27 Aug 1967, *J.A. Steyermark & J.E. Fernández 99757* (US).

***Pilea latifolia*** Wedd. – This species was reported from Venezuela by Killip (1939), who cited a collection (*Cruger s.n.*, K) that lacks detailed locality data but which presumably was made in the Coastal Cordillera.

***Pilea lindeniana*** Wedd. – Killip (1939) reported this species from Mérida, a state record overlooked by Romaniuc Neto (2008).

***Pilea losensis*** Killip – Killip (1936) described this species from a single collection from Colombia (Norte de Santander) and later (Killip, 1939) extended its range to include Venezuela (Aragua). The Venezuelan voucher (*H. Pittier 13984*, US), however, is imperfect and we are uncertain as to its identity. Romaniuc Neto (2008) reported this species from Mérida but we do not know the source of his report. Killip (in sched.) did determine a collection from Mérida (*Steyermark 56365*, US) as *Pilea losensis* but that collection does not agree with the type of *Pilea losensis* and we believe it represents *Pilea miguelii* (see above).

***Pilea microphylla*** (L.) Liebm. – Dorr et al. (2000) reported this species from Trujillo, a state record overlooked by Romaniuc Neto (2008). Romaniuc Neto (2008) considered *Pilea serpyllacea* (Kunth) Liebm. to be a synonym of *Pilea microphylla* but the cyme and leaf shape characters cited by Killip (1936) distinguish the two species. We have not seen material of *Pilea serpyllacea* from Venezuela and the voucher (*Gehriger 258*, US) cited by Killip (1939) has subsessile (versus pedunculate) cymes and appears to be one of the large-leaved forms of *Pilea microphylla* s.l.

***Pilea parietaria*** (L.) Blume – This appears to be the correct name for the Andean species that Killip (1939) recognized as *Pilea rhombea* (L.f.) Liebm. Monro (2001) placed *Pilea rhombea*, described from Mexico, in synonymy under *Pilea parietaria*, described from the West Indies. Monro (2001), however, did not mention a South American element. Killip (1939) thought that *Pilea rhombea* and *Pilea alsinifolia* Wedd. were both confused with the West Indian *Pilea parietaria*. He did acknowledge, however, that all three species were part of the same complex.

***Pilea rhombea*** (L.f.) Liebm. – This species should be deleted from the flora of Venezuela because it is a synonym of *Pilea parietaria* (see above).

## Supplementary Material

XML Treatment for Pilea matthewii

XML Treatment for Pilea miguelii

XML Treatment for Pilea nicholasii

XML Treatment for Pilea nidiae
